# Internet use and physical activity among older adults in China: a multi-dimensional analysis with endogeneity mitigation

**DOI:** 10.3389/fpubh.2026.1862565

**Published:** 2026-08-17

**Authors:** Xiaolu Li, Benle Zhan

**Affiliations:** 1 College of Physical Education, Yan'an University, Yan'an, China; 2 College of Physical Education, Chengdu University, Chengdu, China

**Keywords:** active aging, digital health, effect, internet use, mediation analysis, older adults, physical activity

## Abstract

**Background:**

As population aging accelerates globally, promoting physical activity among older adults has emerged as a central public health challenge. Although internet use has been widely promoted as a potential enabler of healthy aging, its net effects on exercise behavior and the underlying mechanisms remain theoretically ambiguous and empirically underexplored, particularly in large developing economies.

**Methods:**

Using nationally representative microdata from the 2022 China Family Panel Studies (CFPS) covering 6,860 adults aged 60 and above, we examine the association between internet use and three dimensions of exercise behavior: participation, frequency, and intensity. Baseline logistic and OLS regression models are complemented by Karlson–Holm–Breen (KHB) decomposition to assess two hypothesized mediating pathways—learning-oriented health empowerment and entertainment-oriented instant gratification—and by doubly robust treatment-effect estimators (IPWRA and AIPW) to address endogeneity arising from health selection and unobserved confounding.

**Results:**

Internet use is significantly and positively associated with exercise participation (OR = 1.36, *p* < 0.01), while its associations with exercise frequency and intensity are heterogeneous. KHB decomposition reveals that neither learning-oriented empowerment (indirect effect: 1.14% of the total) nor entertainment-oriented instant gratification (3.49%) serves as a statistically significant mediating pathway. Doubly robust estimates confirm a meaningful positive association (IPWRA ATET: +14.9 percentage points; AIPW ATE: +11.0 percentage points). Results are stable across alternative variable definitions, estimation methods, and subsample analyses.

**Conclusion:**

Internet use is associated with exercise participation among older adults in China primarily through direct mechanisms not captured by the learning or entertainment pathways examined. These findings suggest that digital inclusion policies should be integrated into broader public health strategies for active aging, with emphasis on enabling purposeful online engagement rather than passive digital consumption.

## 1 Introduction

Population aging is reshaping public health priorities across the world. Regular physical activity is consistently linked to lower risks of chronic disease, functional decline, and premature mortality, yet exercise participation among older adults remains low in many countries, particularly in low- and middle-income settings. What explains this gap is therefore a question with direct relevance for health policy and health economics.

The rapid spread of internet use has coincided with population aging, raising practical questions about whether digital technologies help or hinder physical activity in later life. There are plausible arguments on both sides. Internet access can widen exposure to health information, enable social connection, and lower barriers to exercise engagement. At the same time, online entertainment may displace time previously spent on physical activity, or reinforce sedentary habits—concerns that are arguably more salient among older adults with declining physical capacity. Empirical work has yet to resolve this tension.

Understanding this tension requires situating internet use within broader theoretical frameworks that explain technology adoption and health behavior change in later life. Three literatures are particularly relevant. First, the digital divide perspective highlights that internet access and use are stratified by age, socioeconomic status, and health status, meaning that the relationship between digital engagement and physical activity cannot be assumed to be uniform across population subgroups (1, 2). Second, theories of social capital suggest that internet use may facilitate exercise not only through information access but also through social connectedness and peer influence—online networks can provide encouragement, accountability, and normative pressure that support health behavior maintenance (20, 21). Third, the theory of planned behavior (TPB) posits that behavioral intentions are shaped by attitudes, subjective norms, and perceived behavioral control (22). Internet use may alter each of these antecedents: it can improve attitudes toward exercise through health information, strengthen subjective norms through online social comparison, and enhance perceived behavioral control by reducing informational barriers to physical activity (3).

The existing literature on internet use and health has concentrated mainly on self-rated health, mental well-being, and healthcare utilization. Physical exercise has received less attention, and the studies that do examine it tend to treat exercise as a single outcome, collapsing participation, frequency, and intensity into one measure. This matters because the decision to exercise at all—and how often or intensively one exercises—likely reflects different behavioral processes, shaped by different constraints and motivations. Treating them as equivalent can mask important variation in how digital technologies affect health behavior in later life.

This study addresses these gaps using microdata from the 2022 China Family Panel Studies (CFPS). We analyze exercise participation, frequency, and intensity as separate outcomes and apply the Karlson–Holm–Breen (KHB) decomposition to examine two mediating pathways: learning-oriented health empowerment and entertainment-oriented instant gratification. While we acknowledge that other mechanisms—particularly social connectedness and digital health information accessibility—may also play important roles, data limitations preclude their formal inclusion in the mediation analysis. We therefore treat these as complementary channels deserving future investigation. To handle potential confounding by health status and unobserved individual traits, we combine rich proxy controls with doubly robust treatment-effect estimators.

We emphasize at the outset that our empirical strategy relies on cross-sectional observational data. While we employ multiple techniques to mitigate endogeneity, including rich proxy controls and doubly robust estimators, we cannot fully rule out unobserved confounding or establish causal identification in the absence of longitudinal or quasi-experimental variation. Our results should therefore be interpreted as conditional associations rather than causal effects, a limitation we address more fully in the Discussion.

By examining multiple dimensions of exercise behavior and testing specific mediating pathways, this study offers a more disaggregated picture of how internet use relates to physical activity in older age. Our findings contribute to the literature by integrating digital divide, social capital, and behavioral theory perspectives within a single empirical framework, and by providing evidence on the relative importance of different mechanisms in a large developing-country setting. The findings have practical relevance for digital inclusion and healthy aging policy.

## 2 Theoretical framework and hypotheses

### 2.1 Internet use and exercise participation among older adults

A growing empirical literature links internet use to greater physical activity among older adults. Studies using CHARLS and CFPS data from China find positive associations with both exercise participation and frequency (4, 18), and similar patterns appear in international research, where digital engagement correlates with higher health awareness and broader participation in health-promoting activities.

Theoretically, this relationship can be situated within the active aging and successful aging frameworks. Active aging, as defined by the World Health Organization, centers on optimizing health, participation, and security throughout the life course. Internet use expands older adults' access to health information, social networks, and community resources, lowering both informational and psychological barriers to physical activity. Recent scholarship has extended Rowe and Kahn's model of successful aging to incorporate digital engagement as a core enabling factor, arguing that online tools now facilitate continued participation and self-management in ways that were previously unavailable to older populations (5).

Empirical evidence further suggests that the effects of internet use are heterogeneous across subgroups, varying by age, gender, and urban–rural residence (19). Nonetheless, the overall direction remains positive: internet use appears to function primarily as a health-enabling technology rather than a risk factor for sedentary behavior among older adults.

Hypothesis 1 (H1).
*Internet use is positively associated with the likelihood of participating in physical exercise among older adults*.

### 2.2 Health empowerment pathway: learning-oriented internet use

Beyond participation per se, scholars increasingly emphasize mechanisms through which internet use affects health behaviors. One central pathway is health empowerment, particularly through learning-oriented online activities. Internet use is associated with broader access to health knowledge, exercise guidance, and disease management information, and this access may help reinforce perceived control and self-efficacy.

Recent studies show that online health information seeking and digital learning improve health literacy and promote proactive health behaviors (6, 7). Systematic reviews of digital behavior change interventions further confirm that tailored information, goal setting, and feedback mechanisms embedded in digital platforms can increase physical activity and reduce sedentary time among older adults, albeit with moderate effect sizes (8, 9).

This empowerment effect may be especially relevant among older adults, for whom declining physical capacity raises the value of better information and behavioral guidance. Online learning can thus serve as a pathway through which internet access translates into changed health behavior.

Hypothesis 2 (H2).
*Learning-oriented internet use positively mediates the relationship between internet use and physical exercise participation among older adults*.

### 2.3 Instant gratification pathway and time preference considerations

In contrast to empowerment mechanisms, concerns persist that entertainment-oriented digital activities may crowd out physical exercise by encouraging sedentary behavior. However, the theoretical and empirical evidence for this instant gratification pathway among older adults is far from conclusive.

Behavioral economics provides critical insights through the lens of time preferences. Studies show that present bias and impatience are associated with poorer health behaviors, including reduced physical activity (10). However, age-related changes in time preferences complicate this relationship. Evidence suggests that while present bias affects the maintenance of physical activity, it does not necessarily impede exercise initiation among older adults (11). Moreover, a balanced time perspective—rather than pure present orientation—is positively associated with higher physical activity levels (3).

Empirical findings on digital entertainment further support this nuanced view. While television viewing is consistently linked to worse functional outcomes and physical decline (12–14), internet-based entertainment and digital gaming show mixed or even neutral effects. Some studies find improvements in balance, mobility, and cognition from interactive digital games (15), whereas others detect no significant adverse or beneficial effects (16).

On balance, the evidence does not support a strong displacement hypothesis for older adults. Digital entertainment and physical activity may simply occupy different time slots, particularly among retirees who face less binding time constraints than working-age adults.

Hypothesis 3 (H3).
*Entertainment-oriented internet use does not significantly mediate the relationship between internet use and physical exercise participation among older adults*.

### 2.4 Digital divide and endogeneity concerns

A final theoretical consideration concerns selection and endogeneity. A substantial literature documents persistent digital divides in later life, with internet users being systematically healthier, younger, and more educated than non-users (1, 2, 17). Health status, cognitive ability, and functional capacity jointly influence both internet adoption and physical activity, raising concerns of reverse causality and omitted variable bias.

While most existing studies acknowledge this issue descriptively, few directly address endogeneity using causal inference techniques. This gap motivates the empirical strategy of the present study, which combines rich proxy controls with doubly robust estimation methods to mitigate selection bias and strengthen causal interpretation.

The theoretical framework identifies two candidate pathways from internet use to exercise: a health empowerment pathway driven by learning-oriented engagement, and a potential displacement pathway from entertainment use. Socioeconomic characteristics, health status, and functional capacity enter as confounders shaping both technology adoption and exercise behavior.

## 3 Data, variables, and empirical strategy

### 3.1 Data

This study uses micro-level survey data from the China Family Panel Studies (CFPS), a nationally representative biennial household panel survey conducted by the Institute of Social Science Survey (ISSS) at Peking University. The CFPS has been implemented since 2010 and employs a multistage, stratified probability sampling design that ensures representativeness at both the national and provincial levels. This study draws on the 2022 wave, which was fielded in 2022–2023 and provides detailed individual-level information on internet use behaviors, physical activity, self-reported health status, chronic disease conditions, functional and cognitive capacity, and a wide range of demographic and socioeconomic characteristics. The CFPS is widely used in empirical research on aging, health, and social inequality in China, and its breadth of variables makes it particularly well suited for examining relationships between digital engagement and health behaviors among older adults. The data are publicly available through the CFPS repository at Peking University (https://www.isss.pku.edu.cn/cfps/).

We restrict the analytical sample to respondents aged 60 years or older. Table 1 presents descriptive statistics for the full sample of 6,860 respondents in this age group with valid information on exercise participation and internet use. The baseline regression sample for the participation analysis is smaller—approximately 2,000 observations—due to listwise deletion of respondents with missing values on any control variable included in the models. This reduction primarily reflects item nonresponse in detailed socioeconomic, health, and cognitive measures, which are not universally administered or completed across all respondents.

**Table 1:** Descriptive statistics.

Variable	Obs	Mean	SD	Min–max
Exercise participation	6,860	0.586	0.493	0–1
Exercise frequency	1,860	5.52	1.38	1–7
Exercise intensity (minutes/week)	1,853	329.47	242.25	4–2,100
Internet use	6,860	0.233	0.423	0–1
Log online time	1,525	4.39	1.08	0–7.08
Importance: work	2,339	3.14	1.44	1–5
Importance: learning	2,354	3.37	1.4	1–5
Importance: entertainment	2,355	3.35	1.31	1–5
Importance: daily life	2,346	2.86	1.51	1–5
Importance: social	2,357	4.21	1.11	1–5
Household savings (IHS)	5,723	0.12	2.24	—
Smoker	6,860	0.18	0.38	0–1
Drinker	6,860	0.1	0.3	0–1

For analyses conditional on exercise participation (Table 2, Panel B), the sample is further restricted to active exercisers, yielding approximately 1,200–1,400 observations depending on the specification Table 3, which sequentially introduces proxy control variables, sample sizes vary across specifications because different covariate groups have different rates of item nonresponse: specifications requiring fewer covariates retain larger samples (up to approximately 5,489 observations), while fully adjusted models yield smaller samples (approximately 1,400 observations). We report the exact sample. In size for each specification in the corresponding table notes and interpret results with reference to these varying analytical samples.

Table 2Internet use and physical exercise among older adults.

**Table:** 

Variables	Exercise (=1)
Panel A. Exercise participation (Logit, Odds Ratios).	
Internet use	1.360^***^ (0.146)
Importance of work-related internet use	0.982 (0.044)
Importance of learning-related internet use	1.102^**^ (0.052)
Importance of entertainment-related internet use	1.049 (0.052)
Importance of daily-life internet use	0.974 (0.040)
Importance of social internet use	0.935 (0.049)
Controls	Yes
Observations	1,999
Pseudo R^2^	0.0886

**Table:** 

Variables	(1) Frequency	(2) ln(Intensity)
Panel B. Exercise frequency and intensity among exercisers (OLS).		
Internet use	−0.215^**^ (0.097)	−0.0468 (0.054)
Importance of work-related internet use	−0.043 (0.040)	−0.036^*^ (0.021)
Importance of learning-related internet use	−0.013 (0.044)	0.0069 (0.022)
Importance of entertainment-related internet use	0.058 (0.050)	0.0755^***^ (0.024)
Importance of daily-life internet use	−0.062 (0.038)	−0.016 (0.020)
Importance of social internet use	0.085^*^ (0.052)	0.0118 (0.028)
Controls	Yes	Yes
Observations	996	995
*R* ^2^	0.052	0.071

Panel A reports odds ratios, average marginal effect (AME) for internet use: 0.0677 (SE = 0.0233, *p* = 0.004), equivalent to a 6.77 percentage-point increase in exercise participation; Panel B reports OLS coefficients. Robust standard errors in parentheses. ^*^p < 0.1, ^**^p < 0.05, ^***^p < 0.01.

**Table 3:** Endogeneity mitigation: proxy controls (Logit, OR).

Variables	(1)	(2)	(3)	(4)	(5)
	Exercise	Exercise	Exercise	Exercise	Exercise
Internet_use	0.971 (0.0763)	2.002^***^ (0.274)	1.908^***^ (0.265)	1.908^***^ (0.265)	5.509 (6.162)
Observations	5,489	1,409	1,408	1,408	71
Pseudo R2	0.0827	0.0957	0.113	0.113	0.283

Odds ratios reported. Standard errors in parentheses. Sample sizes decline across specifications because later columns require additional covariates with higher rates of item nonresponse; the final column relies on early-life health variables available for only a small subsample, and its estimate should be interpreted with caution. ^***^p < 0.01.

To assess whether missing data patterns introduce selection bias, we compared the demographic characteristics of the full sample (*n* = 6,860) against the baseline regression sample (*n* = 2,000). Respondents in the regression sample are slightly younger, more educated, and more likely to reside in urban areas than those excluded due to missing values, consistent with higher item nonresponse among more disadvantaged and older respondents. While this pattern suggests some degree of selection, the direction of bias is unlikely to explain the main findings: if anything, excluding the most disadvantaged respondents would tend to attenuate the estimated association between internet use and exercise participation, making our estimates conservative.

This combination of variables—detailed digital engagement measures, multiple physical activity outcomes, and a broad set of health and socioeconomic covariates—makes CFPS 2022 well suited for the analysis.

### 3.2 Variables

#### 3.2.1 Dependent variables: physical exercise

We examine three complementary measures of physical activity, reflecting both extensive and intensive margins of exercise behavior. First, exercise participation is defined as a binary indicator equal to one if the respondent reports engaging in any physical exercise during the reference period, and zero otherwise. Second, among those who report exercising, exercise frequency is measured on an ordinal scale ranging from 1 to 7, indicating the number of exercise sessions per week. Third, exercise intensity is proxied by total weekly exercise minutes, constructed from reported frequency and duration. Given the right-skewed distribution, we use the natural logarithm of weekly exercise minutes plus one, denoted as ln(weekly exercise minutes + 1). This multi-dimensional measurement strategy allows us to distinguish between the decision to participate in exercise and the intensity of engagement conditional on participation.

#### 3.2.2 Key independent variable: internet use

The main explanatory variable is internet use, defined as a binary indicator equal to one if the respondent reports actively using the internet—such as browsing websites, communicating online, or seeking information—during the survey reference period, and zero otherwise. This measure captures behavioral engagement rather than mere infrastructure access: respondents who reside in areas with internet coverage but do not personally use the internet are coded as non-users. Maintaining this conceptual distinction between internet use and internet access is important, as physical connectivity alone—without active engagement—is unlikely to generate the informational and motivational pathways hypothesized to promote health behaviors. In the CFPS 2022 sample of adults aged 60 and above, approximately 23.3% report using the internet. This rate is consistent with the demographic and geographic composition of the older adult population surveyed, which includes substantial proportions of rural residents and individuals aged 75 and above—two groups with persistently lower rates of digital adoption. Comparable figures have been reported in other nationally representative surveys targeting adults in the same age range in China, suggesting that this rate reflects genuine patterns of digital engagement rather than measurement problems.

To assess robustness and address potential measurement concerns, we further consider alternative indicators of digital engagement, including: logged daily online time, mobile internet use, online learning participation, and a composite index measuring perceived importance of internet use across multiple life domains. These alternative measures serve primarily as robustness checks presented in Section 5.5.1 and Table 4, and are not part of the baseline specification. In Table 2, we additionally present exploratory models that include domain-specific importance indicators (learning, entertainment, daily life, social interaction, and work/income) as separate regressors, with the aim of characterizing heterogeneity across motivational dimensions of internet use. These models should be interpreted as descriptive explorations rather than causal estimates; when multiple importance indicators are included jointly alongside the binary internet use indicator, readers should be aware that multicollinearity may affect the precision and interpretation of individual coefficients. The baseline results in column 1 of Table 2 rely solely on the binary internet use indicator.

**Table 4:** Robustness check (R1): alternative measures of internet use.

Variables	(1) ln online time	(2) Mobile internet use	(3) Online learning	(4) Internet importance index
Panel A. Exercise participation (Logit, Odds Ratios) Dependent variable: exercise participation (1 = Yes)				
ln (1 + Daily online minutes)	0.998 (0.059)			
Mobile internet use (Yes = 1)		1.276^**^ (0.136)		
Online learning (last week)			1.478^*^ (0.343)	
Internet importance index				0.749 (0.191)
Observations	1,253	1,942	1,942	1,942
Pseudo *R*^2^	0.066	0.070	0.069	0.068
**Variables**	**(1) ln online time**	**(2) Mobile internet use**	**(3) Online learning**	**(4) Internet importance index**
Panel B. Exercise frequency (OLS, Exercisers Only) Dependent variable: exercise frequency (1–7)				
ln (1 + Daily online minutes)	0.094 (0.058)			
Mobile internet use (yes = 1)		−0.217^**^ (0.094)		
Online learning (last week)			−0.013 (0.155)	
Internet importance index				0.419^*^ (0.252)
Observations	670	948	948	948
R^2^	0.040	0.034	0.029	0.029
**Variables**	**(1) ln online time**	**(2) Mobile internet use**	**(3) Online learning**	**(4) Internet importance index**
Panel C. Exercise intensity (OLS, Exercisers Only) Dependent variable: ln(Weekly exercise minutes + 1)				
ln (1 + Daily online minutes)	0.085^***^ (0.028)			
Mobile internet use (yes = 1)		−0.050 (0.053)		
Online learning (last week)			−0.028 (0.082)	
Internet importance index				0.114 (0.136)
Observations	669	947	947	947
*R* ^2^	0.073	0.041	0.040	0.040

Odds ratios reported in Panel A. Robust standard errors in parentheses. Panels B and C report OLS estimates among exercisers only. ^*^p < 0.10, ^**^p < 0.05, ^***^p < 0.01.

#### 3.2.3 Control variables

All models include a comprehensive set of control variables capturing individual, household, and regional characteristics. Demographic controls include age (and its square), gender, marital status, urban residence, and years of education. Socioeconomic status is proxied by household savings using an inverse hyperbolic sine (IHS) transformation. To mitigate omitted variable bias related to health selection, we incorporate multiple health-related proxies, including self-rated health, chronic disease indicators, recent hospitalization, and perceived illness severity. Functional ability is captured through indicators of independent daily activities, while cognitive status is measured using standardized word recall and related cognitive tests. Finally, regional fixed effects are included to account for unobserved geographic heterogeneity in infrastructure, healthcare access, and exercise environments.

#### 3.2.4 Descriptive statistics

Table 1 presents descriptive statistics for the main variables, based on the full sample of 6,860 adults aged 60 and above with valid information on internet use and exercise participation.

Approximately 59% of older adults in the sample report participating in any form of physical exercise, indicating substantial heterogeneity in engagement in physical activity at older ages. Among those who exercise, the average weekly exercise frequency is around 5.5 sessions, while the mean exercise duration is approximately 59 minutes per session. Combining frequency and duration, the average weekly exercise intensity amounts to about 330 minutes, though the distribution is highly right-skewed, motivating the use of logarithmic transformations in subsequent analyses.

Regarding digital engagement, about 23% of older adults report using the internet, reflecting persistent digital divides among the older population. Conditional on internet use, average daily online time is modest, but exhibits considerable variation. Measures capturing perceived internet importance across different domains—such as work, learning, entertainment, daily life, and social interaction—suggest that older internet users primarily value digital technologies for information access and social connectivity rather than purely for entertainment purposes.

Control variables indicate substantial socioeconomic and health heterogeneity within the sample. The average age is approximately 68 years, with a roughly balanced gender composition. Educational attainment remains relatively low on average, while notable variation exists in household financial resources. Health-related variables reveal nontrivial prevalence of chronic conditions and functional limitations, underscoring the importance of accounting for health selection when examining the relationship between internet use and physical activity.

Together, these patterns confirm that meaningful variation exists in both digital engagement and physical activity across the sample, supporting the multivariate analyses that follow.

### 3.3 Empirical strategy

#### 3.3.1 Baseline models

We begin by estimating the association between internet use and exercise participation using a logistic regression model, as specified in Equation (1):


$$\mathrm{Pr}\left(Exercise_{i}=1\right)=\text{Logit }\left(\alpha +\beta Internetuse_{i}+X_{i}^{'}\;\gamma +\varepsilon_{i}\right)$$ (1)

where *Exercise*_*i*_ denotes participation in physical exercise, *InternetUse*_*i*_ is the binary indicator of internet use, and $X_{i}^{'}\gamma$ represents the vector of control variables described above. Standard errors are estimated using robust variance estimators.

To examine intensive margins of exercise behavior, we estimate ordinary least squares (OLS) models among exercisers only, as specified in Equation (2):


$$\begin{array}{l} ExerciseOutcome_{i}=\alpha +\beta InternetUse_{i}+{X^{\prime }}_{i}\gamma +\varepsilon_{i} \end{array}$$ (2)

where *ExerciseOutcome*_*i*_ denotes either exercise frequency or logged exercise intensity.

#### 3.3.2 Mechanism analysis

To investigate behavioral mechanisms, we examine potential mediating variables capturing learning-oriented and entertainment-oriented digital use. Mediation analysis is conducted using the Karlson–Holm–Breen (KHB) method, which allows decomposition of total, direct, and indirect effects in nonlinear models while correcting for rescaling bias inherent in logit regressions. This approach enables us to assess whether internet use is associated with exercise behavior primarily through health empowerment channels (e.g., learning and information acquisition) or through instant gratification pathways (e.g., entertainment-oriented use).

Measurement of mediating pathways. The KHB decomposition examines two hypothesized mediating pathways: learning-oriented health empowerment and entertainment-oriented instant gratification. Both mediators are measured using respondents' subjective ratings of the importance of internet use for learning and entertainment, respectively, on ordinal scales. We acknowledge that these perceptual measures capture motivational orientation rather than objective behavioral engagement. The CFPS does not include time-use diaries or logged data on specific online activities, which precludes the use of objective indicators such as hours spent on online learning or entertainment platforms. Consequently, the mediation analysis should be interpreted as testing whether the perceived importance of learning and entertainment mediates the internet–exercise association, not whether objective time spent on these activities does so. This measurement limitation is addressed more fully in the Limitations section.

#### 3.3.3 Addressing endogeneity

Internet use may be endogenous due to reverse causality or unobserved individual traits that jointly affect digital adoption and physical activity. To mitigate these concerns, we adopt a multi-pronged strategy.

First, we progressively augment the baseline model with rich proxy controls capturing health status, functional ability, cognitive capacity, and early-life health, thereby reducing omitted variable bias.

Second, we implement doubly robust treatment effect estimators, including inverse probability weighted regression adjustment (IPWRA) and augmented inverse probability weighting (AIPW). These methods combine propensity score modeling with outcome regression and yield consistent estimates if either the treatment model or the outcome model is correctly specified.

Third, we conduct subsample analyses based on health conditions and functional limitations to assess whether results are driven by selective survival or health-based selection into internet use.

Taken together, these strategies address the main threats to inference while acknowledging the observational nature of the data.

## 4 Empirical results

### 4.1 . Internet use and exercise participation

Table 2 Panel A presents the baseline results on the association between internet use and exercise participation among older adults. After controlling for a comprehensive set of individual, household, and regional characteristics, internet use is found to be significantly and positively associated with the likelihood of engaging in physical exercise. Specifically, older adults who use the internet exhibit a 36.0% higher odds of participating in exercise compared with non-users (OR = 1.360, *p* < 0.01).

This finding is consistent with internet use functioning as a motivational and informational resource—improving health awareness, enabling social engagement, or reducing perceived barriers to exercise. The estimated effect is robust to the inclusion of socioeconomic controls and province fixed effects.

In addition, the results reveal notable heterogeneity across different dimensions of internet use. Internet use oriented toward learning is positively associated with exercise participation (OR = 1.102, *p* < 0.05), indicating that older adults who perceive the internet as an important learning tool are more likely to engage in physical exercise. This pattern is consistent with the notion of digital health empowerment, whereby learning-oriented internet activities may enhance health literacy and promote forward-looking health behaviors.

By contrast, internet use for entertainment, social interaction, or daily life purposes does not exhibit a statistically significant association with exercise participation. These findings suggest that not all forms of internet engagement contribute equally to health-promoting behaviors, highlighting the importance of distinguishing between different motivations and uses of digital technologies among older populations.

### 4.2 . Internet use, exercise frequency, and exercise intensity

While internet use appears to facilitate exercise participation, its relationship with the intensity and regularity of exercise is more nuanced. Table 2 Panel B reports ordinary least squares (OLS) estimates for exercise frequency and exercise intensity, conditional on participation in physical exercise.

The results indicate that, among older adults who already engage in exercise, internet use is associated with a significantly lower frequency of exercise. Specifically, internet users exercise approximately 0.22 fewer times per week compared with non-users (*p* < 0.05). This finding suggests a potential time substitution effect, whereby internet use may crowd out the regularity of physical activity among active individuals.

In contrast, internet use does not exhibit a statistically significant association with overall exercise intensity, measured as the logarithm of weekly exercise minutes. This implies that although internet use may reduce how often older adults exercise, it does not necessarily diminish the total amount of physical activity accumulated over time. One possible interpretation is that older adults who use the internet may compensate for reduced exercise frequency by engaging in longer or more intensive exercise sessions.

Further heterogeneity is observed across different types of internet use. Entertainment-oriented internet use is positively and significantly associated with exercise intensity (*p* < 0.01), suggesting that leisure-related digital activities may coexist with, or even complement, higher-intensity physical activity. This pattern may reflect the influence of digital media content related to exercise, such as online fitness videos or activity tracking applications, although further investigation is required to substantiate these mechanisms.

Taken together, these results point to an asymmetry: internet use is more consistently linked to whether older adults exercise at all than to how often or how intensively they do so among those already active.

### 4.3 Summary of baseline findings

The baseline evidence points to a net positive association of internet use on exercise participation, driven in part by learning-oriented digital engagement, alongside a modest reduction in exercise frequency among active users. These patterns motivate the mechanism and robustness analyses in the sections that follow.

These baseline findings motivate further analysis of the underlying mechanisms and moderating factors through which internet use influences physical activity, which are explored in the subsequent sections.

## 5 Endogeneity concerns

### 5.1 Potential endogeneity concerns

Estimating the causal effect of internet use on exercise participation is complicated by two related problems.

The primary source of endogeneity in this setting is unobserved confounding rather than reverse causality. Latent health capital, functional ability, cognitive capacity, and long-term lifestyle preferences may simultaneously influence both internet adoption and physical activity, generating a spurious positive correlation between the two. Healthier and more cognitively capable individuals may be more likely to use the internet and to exercise independently of any causal relationship between these behaviors. Although reverse causality—whereby more active older adults subsequently adopt internet technologies because of their activity—is conceptually possible in some settings, it is a less plausible primary concern here: exercise participation is unlikely to directly cause internet adoption, given that digital uptake among older adults is predominantly driven by social networks, cognitive resources, and economic factors. The more pressing identification challenge is therefore confounding by unobserved health and socioeconomic characteristics that jointly predict both digital engagement and physical activity.

To address these concerns, this study adopts a multi-pronged endogeneity mitigation strategy, combining (i) rich proxy controls for health, function, cognition, and early-life conditions; (ii) doubly robust treatment-effect estimators; and (iii) subsample analyses based on health and functional status. While these approaches do not claim full causal identification, together they substantially reduce the likelihood that the main findings are driven by endogeneity.

### 5.2 Proxy controls for health, function, and cognition

We first mitigate endogeneity by sequentially adding extensive proxy controls that capture otherwise unobserved determinants of both internet use and exercise participation. Specifically, we augment the baseline logit model with groups of covariates reflecting: Current health status, including self-rated health, chronic disease indicators, hospitalization experience, and illness severity; Functional ability, such as the ability to perform independent outdoor and household activities; Cognitive capacity, measured by word-list test participation and completion; Early-life health conditions, where available, capturing long-term health endowments.

Table 3 reports the results. Across specifications, the estimated odds ratio of internet use remains positive and statistically significant after controlling for health, functional, and cognitive proxies. Notably, the magnitude of the internet-use coefficient is associated with an increase in once health-related variables are included. This pattern is consistent with negative health selection into internet use in this sample: individuals with poorer health—who exercise less on average—may use the internet at comparable or higher rates than healthier peers, possibly because online resources offer particular value for health information seeking and chronic disease management. Under this scenario, unadjusted estimates understate the positive association of internet use on exercise, because the internet-user group is partially composed of less-healthy individuals whose lower exercise rates attenuate the observed difference. Adding health covariates corrects for this selection, yielding larger estimated effects. The final specification yields a much larger point estimate, but the 95% confidence interval is wide and the estimate is statistically imprecise. The reduction in sample size across specifications reflects item nonresponse from different combinations of control variables: each additional covariate excludes observations with missing values on that variable. With fewer observations, standard errors widen and the point estimate becomes sensitive to minor changes in specification. We therefore present the final model as a sensitivity check rather than a primary estimate Overall, these results indicate that the positive association between internet use and exercise participation is not driven by contemporaneous health status, functional ability, or cognitive capacity, alleviating concerns of omitted variable bias from these sources.

### 5.3 Doubly robust treatment effect estimates

To further address selection bias and reverse causality, we employ doubly robust treatment-effect estimators, including Inverse Probability Weighted Regression Adjustment (IPWRA) and Augmented Inverse Probability Weighting (AIPW). These methods jointly model the treatment assignment (internet use) and the outcome (exercise participation), yielding consistent estimates as long as either the treatment model or the outcome model is correctly specified.

Table 5 presents the results. Using IPWRA, we estimate the Average Treatment Effect on the Treated (ATET) and find that internet use is associated with a 14.9 percentage-point increase in the probability of participating in physical exercise. Using AIPW, which estimates the Average Treatment Effect (ATE) for the full population, we find a similarly positive association of approximately 11.0 percentage points. Both estimates are statistically significant at the 1% level. The close agreement between IPWRA and AIPW estimates provides strong reassurance that the main results are robust to selection on observables and are unlikely to be driven solely by endogenous internet adoption. Importantly, these effects are economically meaningful, indicating that internet use is associated with substantial higher levels of exercise participation among older adults.

**Table 5:** Doubly robust estimates of the effect of internet use on exercise participation.

Variables	IPWRA (ATET)	AIPW (ATE)
Internet use	0.149^***^ (0.032)	0.110^***^ (0.031)
Observations	1,408	1,408

Estimates report average treatment effects. IPWRA reports the average treatment effect on the treated (ATET), while AIPW reports the average treatment effect (ATE). Robust standard errors are shown in parentheses. All models control for baseline demographics, health status, functional limitations, cognitive measures, and region fixed effects. ^***^p < 0.01.

### 5.4 Subsample analyses based on health and functional status

As an additional robustness check against reverse causality, we conduct subsample analyses stratified by chronic disease status and functional limitations. The intuition is straightforward: if the observed relationship primarily reflects better underlying health leading to both internet use and exercise, then the internet-use effect should weaken or disappear among less healthy or functionally constrained individuals.

Table 6 reports the results. We find that the positive association between internet use and exercise participation persists among older adults with chronic conditions, with statistically significant effects comparable in magnitude to those observed in healthier subsamples. This finding weakens the reverse-causality explanation that only healthier individuals drive the baseline results. By contrast, the association between internet use and exercise participation becomes statistically insignificant among individuals with severe functional limitations. This pattern is consistent with behavioral constraints: even if internet use is associated with better information access or greater motivation, physical limitations may prevent these individuals from translating such benefits into actual exercise behavior. Rather than undermining the main results, this heterogeneity supports their plausibility.

**Table 6:** Endogeneity mitigation: subsample checks (Logit, OR).

Variables	(1)	(2)	(3)	(4)
	Exercise	Exercise	Exercise	Exercise
Internet_use	2.109^***^ (0.417)	1.746^***^ (0.339)	0.921 (0.502)	2.045^***^ (0.297)
Observations	701	707	245	1,163
Pseudo R2	0.126	0.116	0.134	0.0879

Odds ratios reported. Subsamples by chronic conditions and functional limitation (proxy). ^***^p < 0.01.

Taken together, the three complementary approaches—proxy controls, doubly robust estimators, and health-stratified subsamples—consistently return a positive and meaningful association between internet use and exercise participation. The convergence of results across these strategies reduces the plausibility of a purely selection-driven explanation.

### 5.5 Robustness checks

We conducted four sets of robustness checks: alternative measures of internet use, alternative exercise outcome definitions, alternative estimation methods, and subsample analyses. The main finding—a positive association between internet use and exercise participation—holds across all of them, with effect size and significance varying across specifications in ways that illuminate the heterogeneity of the relationship.

#### 5.5.1 Robustness check R1: alternative measures of internet use

Table 4 presents results using alternative indicators of internet use, including daily online time, mobile internet use, recent online learning participation, and a composite index of perceived internet importance.

For exercise participation, replacing the binary indicator of internet use with daily online time yields a statistically insignificant association (OR = 0.998), suggesting that the sheer amount of time spent online is not the key driver of the participation effect. In contrast, mobile internet use is positively associated with exercise participation (OR = 1.276, *p* < 0.05). This finding is consistent with the overall positive baseline association and may reflect the fact that mobile internet users—who can go online via smartphones regardless of location—are more actively and habitually engaged with digital content than those relying solely on fixed-line connections. Similarly, online learning participation in the previous week is associated with a higher probability of exercising (OR = 1.478, *p* < 0.10), lending support to the notion that cognitively oriented and goal-directed internet use is more conducive to health-promoting behaviors.

By comparison, the internet importance index, which captures general subjective valuation of internet use across multiple domains, shows no statistically significant association with exercise participation. This finding suggests that behavioral engagement with specific online activities may matter more than overall attitudinal importance.

Among exercisers, results for exercise frequency and exercise intensity further underscore this distinction. Daily online time is positively associated with exercise intensity (β = 0.085, *p* < 0.01), but not with participation or frequency. Mobile internet use is negatively associated with exercise frequency (β = −0.217, *p* < 0.05), while the internet importance index is positively related to frequency (β = 0.419, *p* < 0.10). Taken together, these findings suggest that different dimensions of internet use may exert heterogeneous effects on how often and how intensively older adults exercise.

#### 5.5.2 Robustness check R2: alternative definitions of exercise outcomes

Table 7 reports robustness checks using alternative binary definitions of exercise behavior. When defining high-frequency exercise as engaging in physical activity at least three times per week, internet use remains positively associated with exercise (OR = 1.210, *p* < 0.10). However, no statistically significant association is found when defining high-intensity exercise as having exercise intensity above the median. These results indicate that internet use among older adults is more strongly related to the decision to exercise regularly, rather than to achieving higher levels of exercise intensity. This pattern is consistent with a behavioral interpretation in which internet use facilitates initiation and maintenance of routine physical activity, rather than pushing individuals toward more strenuous exercise.

**Table 7:** Robustness check: alternative binary definitions of exercise.

Variables	(1)	(2)
	High frequency (≥3/week)	High intensity (≥median)
Internet use	1.210^*^ (0.131)	0.993 (0.121)
Observations	1934.000	1933.000
Pseudo R^2^	0.068	0.048

Odds ratios reported. Robust standard errors in parentheses. All models control for individual, household, and regional characteristics. ^*^p < 0.1.

#### 5.5.3 Robustness check R3: alternative estimation methods

To examine whether the baseline results are sensitive to model specification, we estimated a Probit model for exercise participation and count data models for exercise frequency (Tables 8, 9).

**Table 8:** Robustness: probit model for exercise participation.

Variables	Probit
Internet use	0.154^**^ (0.066)
Observations	1,942.000
Pseudo R^2^	0.070

Probit coefficients reported. ^**^p < 0.05.

**Table 9:** Models robustness: count for weekly exercise frequency (freq0).

Variables	(1)	(2)
	Poisson	Negative binomial
Internet use	0.084 (0.058)	0.074 (0.063)
Observations	1,934.000	1,934.000

Coefficients reported. Robust standard errors in parentheses. All models include the same set of controls as the baseline specification.

The Probit results confirm the baseline Logit findings, with internet use remaining positively and significantly associated with exercise participation (coefficient = 0.154, *p* < 0.05). This consistency across nonlinear probability models suggests that the main results are not driven by the choice of link function.

For exercise frequency measured as a count variable (including non-exercisers), both Poisson and Negative Binomial models yield positive but statistically insignificant coefficients for internet use. This finding aligns with earlier results showing that internet use is more robustly related to participation than to the absolute number of exercise sessions, and suggests limited sensitivity of frequency outcomes to model choice.

#### 5.5.4 Robustness check R4: subsample analyses

Finally, Table 10 reports subsample analyses to assess whether the baseline association is driven by specific demographic groups. The positive association between internet use and exercise participation remains statistically significant among urban residents (OR = 1.499, *p* < 0.01) and men (OR = 1.554, *p* < 0.01), but is weaker and statistically insignificant among rural residents and women. Among individuals aged 65 and above, the estimated coefficient remains positive but does not reach conventional levels of statistical significance.

**Table 10:** Robustness: Subsample analyses for exercise participation.

Variables	(1)	(2)	(3)	(4)	(5)
	Age ≥ 65	Urban	Rural	Male	Female
Internet use	1.237 (0.172)	1.499^***^ (0.216)	1.075 (0.175)	1.554^***^ (0.229)	1.072 (0.165)
Observations	1,175.000	1,142.000	800.000	1,056.000	886.000
Pseudo R^2^	0.073	0.041	0.037	0.091	0.066

Odds ratios reported. Robust standard errors in parentheses. Each model includes the same set of controls as the baseline specification. ^***^p < 0.01.

These results suggest that the exercise-promoting effects of internet use are more pronounced among groups with relatively better digital access and greater baseline opportunities for physical activity. Importantly, the direction of the association remains consistent across all subsamples, alleviating concerns that the main findings are driven by a single demographic group.

Across all four robustness checks, internet use remains positively associated with exercise participation, while its associations with frequency and intensity are more variable. The pattern across alternative internet use measures suggests that the type and purpose of digital engagement matter more than aggregate time online.

## 6 Mechanism analysis: mediation pathways

Table 11 reports the results of the KHB decomposition examining two hypothesized mediation pathways through which internet use may influence exercise participation among older adults: learning-oriented empowerment and instant gratification. By decomposing the total effect into direct and indirect components while accounting for rescaling bias inherent in nonlinear models, the KHB approach allows a rigorous assessment of whether these pathways meaningfully explain the observed association.

**Table 11:** KHB decomposition of mediation pathways between internet use and exercise participation.

Variables	Reduced model	Full model	Difference
Panel A. Learning-oriented empowerment pathway			
Internet use	0.291^***^ −0.103	0.288^***^−0.103	0.003 −0.005
Controls	Yes	Yes	Yes
Mediator included	No	Yes (imp_learn)	–
Observations	2028	2028	2028
Pseudo R^2^	0.07	0.07	–
**Statistic**	**Value**		
KHB summary statistics			
Confounding ratio	1.012		
Percentage mediated (%)	1.14		
Rescaling factor	1.002		
**Variables**	**Reduced model**	**Full model**	**Difference**
Panel B. Instant gratification pathway.			
Internet use	0.260^**^ −0.104	0.251^**^−0.104	0.009 −0.007
Controls	Yes	Yes	Yes
Mediator included	No	Yes (imp_entertain)	—
Observations	1,967	1,967	1,967
Pseudo *R*^2^	0.07	0.07	—
**Statistic**	**Value**		
KHB summary statistics			
Confounding ratio	1.036		
Percentage mediated (%)	3.49		
Rescaling factor	1.003		

Coefficients reported are log-odds. Robust standard errors in parentheses. Reduced models exclude the mediator; full models include the corresponding mediator. The “Difference” column reports the indirect (mediated) effect net of rescaling. All models control for smoking, drinking, household net assets (IHS), gender, age (and age squared), marital status, urban residence, education, and region fixed effects. ^**^p < 0.05, ^***^p < 0.01.

### 6.1 Learning-oriented empowerment pathway

Table 11 Panel A presents the results for the learning-oriented empowerment mechanism, proxied by the importance attached to internet-based learning (*imp_learn*). Internet use is positively and significantly associated with exercise participation in both the reduced model (β = 0.291, SE = 0.103, *p* < 0.01) and the full model controlling for the mediator (β = 0.288, SE = 0.103, *p* < 0.01). The estimated indirect effect is small and statistically insignificant (Diff = 0.003, SE = 0.005), accounting for only 1.14% of the total effect according to the KHB summary statistics.

One interpretation is that health information gained online does not translate directly into changed behavior for most older adults, given constraints from physical capacity, mobility, and established routines. Learning-oriented internet use may raise awareness without being sufficient to shift exercise habits. In our data, however, this perceived importance does not translate into a statistically significant indirect effect.

### 6.2 Instant gratification pathway

Table 11 Panel B examines the instant gratification mechanism, proxied by entertainment-oriented internet use (*imp_entertain*). Internet use remains positively associated with exercise participation, with only a modest decline in the coefficient after including the mediator (from β = 0.260 to β = 0.251, both *p* < 0.05). The estimated indirect effect is again statistically insignificant (Diff = 0.009, SE = 0.007), explaining 3.49% of the total effect.

This null result is consistent with the view that entertainment use among older adults tends to be embedded within existing daily routines rather than replacing discrete physical activity time. It is also consistent with evidence that older adults show relatively lower present bias compared to younger cohorts, making hedonic displacement of exercise a less likely mechanism in this population (3, 11).

### 6.3 Overall interpretation

It is important to emphasize that the absence of significant indirect effects does not invalidate the observed association between internet use and exercise participation. Rather, it indicates that the mechanisms examined here do not explain that association, and that additional pathways should be investigated in future research. The KHB results indicate that neither pathway accounts for a meaningful share of the total internet-use effect on exercise participation. The dominant channel appears to be direct—possibly reflecting social connectedness, motivation, or resource access effects that the two mediators tested here do not adequately capture. These mechanisms remain theoretically plausible but could not be directly examined with the available CFPS measures. Identifying these residual pathways is a priority for future research.

## 7 Discussion and policy implications

### 7.1 Discussion

This study examined the conditional association between internet use and exercise behavior among adults aged 60 and above, using cross-sectional data from the CFPS 2022. The main finding—a positive association between internet use and exercise participation—holds across a range of specifications and endogeneity mitigation approaches. Effects on exercise frequency and intensity are more variable and generally weaker. Thus, the null mediation findings should not be read as evidence against the main association; they point to the existence of other, unmeasured mechanisms.

The baseline results indicate that older adults who use the internet are significantly more likely to engage in physical exercise. This positive association persists when internet use is measured using alternative indicators—such as mobile internet use and online learning participation—and when participation is modeled using both logit and probit specifications. These findings align with a growing body of literature suggesting that digital engagement can support healthier lifestyles in later life by improving access to health information, expanding social networks, and reducing barriers to participation in health-promoting activities.

The pattern across the intensive margin outcomes suggests that internet use is more strongly linked to whether older adults begin exercising than to how much they exercise once they do. This is consistent with research showing that digital tools facilitate initial motivation and health awareness but have weaker effects on sustained behavioral maintenance (8).

The KHB decomposition results add nuance to this picture. The learning-oriented empowerment pathway shows a small and statistically insignificant indirect effect, is not the primary channel empirically supported in this study. The entertainment pathway is similarly insignificant, indicating that digital leisure does not meaningfully crowd out physical activity. Behavioral economics offers one explanation: older adults tend to show lower present bias than younger cohorts, which may reduce the extent to which hedonic digital activities compete with health-promoting behavior (11).

The robustness analyses further strengthen the credibility of the findings. The positive association between internet use and exercise participation holds across alternative outcome definitions, including binary indicators of high exercise frequency and high intensity. Subsample analyses reveal notable heterogeneity: the effect of internet use is particularly strong among urban residents and male older adults, while it is weaker or statistically insignificant among rural residents and females. These differences likely reflect disparities in digital infrastructure, health resources, and social norms surrounding exercise, highlighting the importance of contextual factors in shaping the digital–health nexus.

Addressing endogeneity is central to the empirical contribution of this study. We combined three strategies—progressive proxy controls, doubly robust estimators, and health-stratified subsamples—to reduce the plausibility of selection-driven findings. Results are qualitatively consistent across all three approaches. Nevertheless, we emphasize that these methods mitigate rather than eliminate endogeneity concerns. The cross-sectional data structure precludes full causal identification; unobserved confounders, time-invariant individual heterogeneity, and potential reverse causality cannot be fully ruled out. Our estimates should therefore be interpreted as conditional associations that are informative about the relationship between internet use and exercise behavior, but not as causal effects. This limitation should be addressed in future work with longitudinal or quasi-experimental designs.

The findings point to several policy-relevant considerations that go beyond general recommendations for digital inclusion.

Urban–rural equity. Expanding meaningful internet access among older adults may have benefits for physical activity, but simply extending broadband coverage to rural areas is unlikely to be sufficient. Targeted interventions should integrate digital literacy training with existing community health programs. For example, village health stations could serve as dual-purpose hubs where older adults receive both basic health screenings and guided instruction on using smartphones to access exercise-related content. Subsidized low-cost terminals and technical support hotlines tailored to older users could address the “device + skill” barrier that complements infrastructure gaps.

Gender-sensitive programming. The weaker effects among women suggest that digital health interventions need to be designed with attention to gendered time constraints and social norms. Community-based programs that combine online exercise video libraries with offline female-only exercise groups may be more effective than purely digital approaches. Scheduling flexibility—recognizing that many older women continue to bear primary caregiving responsibilities—and content tailored to home-based, low-impact exercises may help overcome barriers to public exercise participation. Peer support networks facilitated through social media or messaging apps could also provide normative reinforcement in a format that respects women's preferences for private or small-group settings.

Community health service integration. Primary care providers and community health workers are well positioned to bridge the digital–physical activity gap. Integrating brief digital health prescriptions—for example, recommending specific exercise apps, online tai chi classes, or walking group coordination platforms—into routine health consultations for older adults could leverage the trust and authority of medical professionals to promote both internet use and physical activity. Community health centers could further serve as physical spaces where older adults access devices, receive training, and participate in supervised group exercise, creating an ecosystem where digital tools and offline resources reinforce each other.

Type and purpose of digital engagement. Neither entertainment-oriented nor learning-oriented use shows a statistically significant mediating pathway, although the latter accounts for a slightly larger percentage of the total effect. Programs encouraging older adults to use the internet purposefully—for health information, peer support, or structured online activity—are more likely to produce physical activity benefits than passive content consumption. However, policymakers should avoid framing digital entertainment as inherently harmful; our results show no evidence of crowding out, suggesting that a balanced approach integrating digital leisure with physical and social activities is both realistic and acceptable for older adults.

Age-adaptive approaches. For adults aged 65 and above, simplified interfaces, larger text and icons, voice-assisted navigation, and family-mediated digital support may be necessary to ensure that internet access translates into meaningful health engagement. Interventions should also account for declining functional capacity by emphasizing low-impact, home-based exercise options that can be discovered and guided through digital platforms.

More broadly, as internet adoption continues to spread among older cohorts, understanding how digital engagement interacts with health behavior will become increasingly relevant for public health. Integrating digital tools into active aging policy is no longer a marginal concern.

In summary, internet use is robustly associated with exercise participation among older adults in China, primarily at the extensive margin rather than through changes in exercise intensity or frequency. The direct channel dominates; neither learning-oriented empowerment nor entertainment-oriented instant gratification accounts for a meaningful indirect effect. The association is concentrated among urban residents and men, reflecting underlying disparities in digital infrastructure, social norms, and health resource access. Future research with longitudinal or quasi-experimental designs and qualitative methods would help clarify the mechanisms behind this association.

This study has several limitations that should be considered when interpreting the findings.

First, the analysis relies on cross-sectional data from a single wave of the CFPS which precludes causal identification. While we employ multiple strategies to mitigate endogeneity—including rich proxy controls for health, function, and cognition, as well as doubly robust treatment-effect estimators—we cannot fully rule out unobserved confounding, time-invariant individual heterogeneity, or reverse causality. The doubly robust estimates (IPWRA and AIPW) assume no unmeasured confounders conditional on the observed covariates; if important determinants of both internet use and exercise behavior remain unobserved, the estimated associations may still be biased. Future research using longitudinal panel data, natural experiments, or instrumental variable approaches is needed to strengthen causal inference.

Second, the measures of internet use and physical activity are based on self-reported survey responses, which may be subject to recall bias and social desirability effects. Older adults may overreport exercise participation and underreport sedentary internet use, potentially attenuating the estimated associations. Objective measures—such as device-tracked physical activity or logged internet usage time—would improve measurement precision but are not available in the CFPS.

Third, the mediating pathways examined in the KHB decomposition rely on subjective measures of perceived internet importance (learning-oriented and entertainment-oriented) rather than objective indicators of online time or specific digital activities. While these perceptual measures capture motivational orientation, they do not directly quantify behavior. Consequently, the null mediation results should be interpreted cautiously: they indicate that the perceived importance of learning and entertainment does not mediate the association, but do not rule out the possibility that objective online learning time or specific entertainment behaviors might operate through different mechanisms.

Fourth, the sample size varies considerably across specifications due to listwise deletion of observations with missing values on control variables. The baseline participation regression includes approximately 2,000 observations, while proxy-control specifications range from approximately 1,400 to 5,489 observations depending on the covariates required. Although we report all sample sizes transparently, differential attrition across specifications may introduce selection bias if missingness is related to unobserved determinants of the outcome.

Fifth, the CFPS does not include direct measures of social connectedness, peer interaction, or digital health information accessibility—mechanisms that theoretical frameworks (social capital theory, theory of planned behavior) suggest may be important. Our inability to test these pathways formally represents a gap that future data collection efforts should address. Additionally, the observed negative health selection into internet use among frail older adults may partly reflect health-seeking behavior: less healthy individuals may go online more frequently to search for health information or manage chronic conditions, while at the same time being advised by health professionals to limit strenuous physical activity. This combination can mechanically produce lower baseline exercise participation among internet users, which our proxy controls attempt to address but cannot fully disentangle without detailed clinical records or longitudinal health trajectories.

Sixth, the generalizability of our findings to other countries and cultural contexts is uncertain. The digital landscape, exercise norms, and health infrastructure in China differ substantially from those in high-income Western settings. Replication in other populations would strengthen the external validity of the results.

## Data Availability

The original contributions presented in the study are included in the article/supplementary material, further inquiries can be directed to the corresponding author.
